# The Antioxidant Activity of Pistachios Reduces Cardiac Tissue Injury of Acute Ischemia/Reperfusion (I/R) in Diabetic Streptozotocin (STZ)-Induced Hyperglycaemic Rats

**DOI:** 10.3389/fphar.2018.00051

**Published:** 2018-02-06

**Authors:** Rosanna Di Paola, Roberta Fusco, Enrico Gugliandolo, Ramona D'Amico, Michela Campolo, Saverio Latteri, Arianna Carughi, Giuseppina Mandalari, Salvatore Cuzzocrea

**Affiliations:** ^1^Department of Chemical, Biological, Pharmaceutical and Environmental Sciences, University of Messina, Messina, Italy; ^2^Department of General Surgery, Cannizzaro Hospital, University of Catania, Catania, Italy; ^3^American Pistachio Growers, Fresno, CA, United States

**Keywords:** animal model, anti-inflammation, antioxidants, cardiac ischemia, hyperglicemia

## Abstract

Diabetes mellitus is an important risk factor for the development of heart pathology. Myocardial infarction is the cause of death occurring after prolonged ischemia of the coronary arteries. Restoration of blood flow is the first intervention against heart attack, although the process of restoring blood flow to the ischemic myocardium could cause additional injury. This phenomenon, termed myocardial ischemia-reperfusion (MI-R) injury, is characterized by the formation of oxygen radicals. Pistachios have significant glucose- and insulin-lowering effects and can improve the inflammatory contest by downregulating both the expression and the circulating levels of several metabolic risk markers. The monocyte/macrophage cell line J774 was used to assess the extent of protection by natural raw (NP) and roasted salted (RP) pistachios against lipopolysaccharide (LPS)-induced inflammation. Moreover, antioxidant activity of NP and RP was assessed in an *in vivo* model of paw edema in rats induced by carrageenan (CAR) injection in the paw. This study evaluates the antioxidant properties of pistachios on the inflammatory process associated with myocardial ischemia/reperfusion injury (I/R) in diabetic rats. Rats were pre-treated with either NP or RP pistachios (30 mg/kg) 18 h prior to the experimental procedure.

**Results:** Here, we demonstrated that treatment with NP reduced myocardial tissue injury, neutrophil infiltration, adhesion molecules (ICAM-1, P-selectin) expression, proinflammatory cytokines (TNF-α, IL-1β) production, nitrotyrosine and PAR formation, NF-κB expression and apoptosis (Bax, Bcl-2) activation. This data clearly showes modulation of the inflammatory process, associated with MI-R injury, following administration of pistachios.

## Introduction

Heart physiology suffers from the occurrence of risk factors such as hypertension and dysmetabolic syndromes, which may aggravate concomitant heart insults. Among dysmetabolic factors, diabetes is an important risks for development of heart pathologies. It is a cause of vascular and cardiac dysfunction itself, through formation of reactive-oxygen species (ROS) and can also compromise other cardiovascular pathologies. It is notorious that oxidative stress is a component of diabetes and its complications (Cakatay et al., 2000; Haak, 2000; Obrosova and Fathallah, 2000; Pieper and Siebeneich, 2000). Cardiovascular disease is one of the major complication of diabetes, which can lead to death in these patients (Kwasny et al., 2017). In the last few years, it has been shown that hyperglycaemia should be one of the causes of the cardiovascular alterations associated with diabetes (Silaghi et al., 2015). Therefore, hyperglycaemia is a significant predictive risk factor for myocardial infarction and hypertension in patients with and/or without diabetes (Lip et al., 2017). It is not unexpected that an increase in the glucose level can produce arrhythmias with QT interval prolongation, fatal in case of infarction, and increases coronary constriction (Nogueira et al., 2008). Hyperglycaemia causes *per se* oxidative stress (Pieme et al., 2017). A common feature to cellular damage caused by hyperglycaemia is an up-regulated production of ROS, which is a main cause of the clinical complications associated with diabetes. Another study has clearly showed that an amplified production of ROS is responsible for the hyperglycaemia-induced electrophysiological alterations (Fernandez-Ruiz et al., 2017). Experimental studies have displayed that treatment with antioxidants reduces the vascular dysfunction and oxidative stress associated with experimental diabetes (Paterniti et al., 2017). The effects of the intake of nuts on insulin levels and blood glucose was assessed (Wien et al., 2010), but less is known on the action of pistachios intake (Sari et al., 2010). Pistachios possess considerable antioxidant properties, attributable to the presence of luteolin and polyphenolic compounds, including flavonoids and anthocyanins. Amongst tree nuts, pistachios have the highest content of phytosterols, including stigmasterol, campesterol, and β-sitosterol (Ros, 2010). This full set of macronutrients and micronutrients means that pistachio nuts should have a positive effect on the inflammatory and oxidative state (Martorana et al., 2013). We have previously demonstrated that bioactives from pistachios were bioaccessible and potentially available for absorption during simulated human digestion (Mandalari et al., 2013). Clinical studies showed that antioxidants in pistachios raise blood levels of lutein, beta-carotene and gamma tocopherol (Kay et al., 2010). Clinical data also demonstrate that chronic consumption of pistachios in patients reduces insulin levels and glucose, thus improving insulin resistance and other metabolic and inflammatory risk markers (Hernandez-Alonso et al., 2014). This study was designed to evaluate the antioxidant effect of natural raw (NP) and roasted salted (RP) pistachios on cardiac tissue injury of acute ischemia/reperfusion (I/R) in diabetic streptozotocin (STZ)-induced hyperglycaemic rats.

## Materials and methods

### Animals

Male Sprague-Dawley rats weighing approximately 200 g were acquired from Charles River (Calco, Italy). The animals were placed in an animal facility under the best conditions required for their housing. Rats were located in stainless steel cages in a room kept at 22 ± 1°C with a 12-h dark, 12-h light cycle. The animals were familiarized to their setting for 2 week and had access to rodent standard diet and water *ad libitum* (Fusco et al., 2017). The research was approved by the University of Messina Review Board for the care of animals and was carried out in agreement with regulations in USA, Europe and Italy. All animal experiments comply with the ARRIVE guidelines.

### Reagents

STZ and phenyl-methyl sulfonyl fluoride were obtained from Sigma-Aldrich (St. Louis, MO). All chemicals were procured from DBA (Milan, Italy). Pistachios were provided by the American Pistachio Growers (CA, US).

### Induction of diabetes

After 12 h fasting, animals received a single 60-mg/kg intravenous (i.v.) inoculation of STZ in 10 mM of sodium citrate buffer (1 mL/kg dose volume). Control (sham, non-diabetic) animals received citrate buffer (vehicle) alone. After 24 h, animals with blood glucose levels >250 mg/dl were considered diabetic, and randomly divided in groups of 10 each. All experiments were performed 60 days following induction of diabetes. We confirmed diabetic condition by evaluation of glucose levels daily by blood glucose meter (Accu-Check Active®, Roche Diagnostic, Milan, Italy).

**Table:** 

**Experimental groups**	**Blood glucose**		
	**Day 0**	**Days 15**	**Days 60**
Sham	122 ± 3.2	135 ± 2.5	128 ± 3.1
I/R	135 ± 2.8	462 ± 3.0	458 ± 2.7
Raw pistachios	124 ± 2.6	454 ± 2.1	450 ± 2.5
Roasted pistachios	130 ± 2.1	465 ± 2.8	463 ± 2.6

### Experimental groups

After 60 days following induction of diabetes rats were randomly assigned into different groups as described below.

1. *STZ-hyperglycaemic rats*: *Ischemia/reperfusion* + *vehicle group:* Diabetic rats were subjected to coronary artery occlusion (30 min) followed by reperfusion (2 h) (*N* = 10) (*n* = 10);
2. *STZ-hyperglycaemic Ischemia/reperfusion rats pre-treated with natural raw pistachios*: Diabetic rats were treated with NP (30 mg/kg) administrated orally (*n* = 10) 18 h prior to the experimental procedure;
3. *STZ-hyperglycaemic Ischemia/reperfusion rats pre-treated with roasted salted pistachios*: Diabetic rats were treated with RP (30 mg/kg) administrated orally (*n* = 10) 18 h prior to the experimental procedure;
4. *Sham* + *saline group:* Rats were subjected to identical surgical procedures except for coronary artery occlusion shock and were kept under anaesthesia for the duration of the experiment (*N* = 10);
5. *Sham* + *natural raw pistachios group:* Identical to sham-operated rats except for oral administration of NP (30 mg/kg) 18 h prior to the experimental procedure; (*N* = 10)
6. *Sham* + *roasted salted pistachios group:* Identical to sham-operated rats except for the oral administration of RP (30 mg/kg) 18 h prior to the experimental procedure; (*N* = 10);

The pistachios dose to be administered (30 mg/kg) was chosen on the basis of previous *in vivo* studies using almond skin (Mandalari et al., 2011). The pistachios time of administration was chosen on the basis of previous studies on the bioaccessibility of pistachio polyphenols, xanthophylls, and tocopherols during simulated human digestion (Mandalari et al., 2013).

### The left anterior descending artery (LAD) ligation: surgical procedures

Animals were anesthetized with thiopentone sodium (120 mg/kg i.p.). Anaesthesia was provided by additional injections of thiopentone sodium as required. After trachea cannulation, the animals were ventilated with a Harvard ventilator (concentration of inspired oxygen: 30%; 70 strokes/min, tidal volume: 8–10 ml/kg). Body temperature was maintained at 37 ± 1°C with the support of a rectal thermometer attached to a Harvard Apparatus™ Homeothermic Blanket Systems with Flexible Probe (Edenbridge, Kent, UK). Thoracotomy was performed at the fifth intercostal space in all rats, the pericardium was removed and a silk thread 6-0 was placed around the left descending coronary artery approximately 1 to 2 mm below its origin. A little silicon ring was introduced in the silk thread under the knot to simplify the successive elimination of the suture. Then, in order to reduce heart displacement, the chest was closed by a silk suture, taking care to consent the ends of the coronary suture threads emerging from the surgical wound. Rats were permitted to equilibrate for 20 min. Ischaemia was induced as described before (Di Paola et al., 2016).

### Quantification of myocardial tissue injury

At the end of the 2 h reperfusion period, the LAD was re-occluded, and 1 mL of Evans blue dye (2% wt/vol) was injected into the animal via the jugular vein. The Evans blue dye stains the tissue so that the non-perfused vascular (occluded) tissue remains uncolored.

The unstained myocardium was incubated for 30 min at 37°C in TTC (1% in 0.1 mol/L phosphate buffer, pH 7.4). The non-infarcted myocardium was deep red, in contrast to the pale of the infarcted myocardium (Zhang et al., 2017).

All animals were sacrificed by an overdose of anesthetic and the heart excised. After removal of the right ventricle, the heart was analyzed as described before (Di Paola et al., 2016).

### Light microscopy

Heart biopsies were taken, from the left ventricle, at 2 h after reperfusion. The heart samples were fixed in formaldehyde solution (10% in phosphate buffered saline) at room temperature, dehydrated by graded ethanol and embedded in Paraplast (Sherwood Medical, Mahwah, NJ). Heart sections (thickness 7 μm) were deparaffinised with xylene stained with haematoxylin/eosin and examined by light microscopy (Dialux 22 Leitz). All histological studies were executed in a blinded manner. The scoring method was chosen based on previous studies using the following criteria: 0, no damage; 1 (minor), focal swelling and necrosis of the myocytes; 2 (severe), necrosis with evidence of neutrophil infiltration in the myocytes; 3 (major), necrosis with massive neutrophil infiltration.

### Immunohistochemical localization of TNF-α, IL-1β, ICAM-1, P-selectin, nitrotyrosine, Par, Bax, and Bcl-2

At 2 h after reperfusion, the heart samples were fixed in 10% buffered formaldehyde and 7 μm sections were prepared from paraffin embedded tissues. After deparaffinization samples were incubated as describes before (Di Paola et al., 2016). The sections were incubated overnight with primary anti-P-selectin antibody, anti-ICAM-1 antibody, antinitrotyrosine antibody, anti-poly (ADP-ribose) antibody, anti-PAR antoboby, anti-Bax antibody, with anti-Bcl-2 antibody, anti TNF-α antibody, anti-IL-1β rabbit polyclonal antibody (1:500 in PBS, v/v) or with control solutions. Controls included buffer alone.

Immunohistochemical images were collected using a Zeiss microscope and Axio Vision software. For graphic display of densitometric analyses, positive staining intensity (brown) was measured by computer-assisted color image analysis (Leica QWin V3, UK).

### Immunofluorescence for CD45, TNF-α, IL-1β, ICAM, P-selectin, nitrotirosine, and PARP

Tissue sections were incubated with one of the next primary antibodies: anti-CD45 (1:100, Santa Cruz), anti- TNF-α (1:100, Santa Cruz Biotechnology), anti- IL-1β (1:100, Santa Cruz Biotechnology), anti-P-selectin antibody (Santa Cruz Biotechnology), anti-ICAM-1 antibody (Santa Cruz Biotechnology), antinitrotyrosine antibody(Santa Cruz Biotechnology), anti-poly (ADP-ribose), anti-PAR antibody in a humidified chamber at 37°C overnight. Sections were washed with PBS and then incubated with either FITC-conjugated anti-mouse Alexa Fluor-488 antibody (1:2,000, Molecular Probes, UK) or Texas Red-conjugated anti-rabbit Alexa Fluor-594 antibody (1:1,000, Molecular Probes) or FITC-conjugated anti-goat Alexa Fluor-488 antibody (1:2,000, Molecular Probes, UK) for 1 h at 37°C. Sections were later cleaned and nuclei stained with 4′,6′-diamidino-2-phenylindole (2 μg/ml in PBS; Hoechst, Frankfurt; Germany). Each tissue section was analyzed and acquired using a Leica DM2000 microscope. The images were digitalized at 8 bits resolution into a range of 2560 × 1920 pixels. Optical sections of fluorescence specimens were acquired using a HeNe laser (543 nm), a laser UV (361–365 nm) and an argon laser (458 nm) at a 1-min, 2-s scanning speed with up to 8 averages; 1.5-μm sections were geted using a pinhole of 250. Brightness and contrast were both calculated by studying the most brightly labeled pixels and enforcing settings that elicited clear visualization of structural details while keeping the highest pixel intensities approximately at 200. The same settings were utilized for all images obtained from the other samples that had been processed in parallel. Digital images were picked and Figure montages settled using Adobe Photoshop CS6 (Adobe Systems; Milan Italy).

#### Western blot analysis for NF-κB P65 and ikB-Alpha

In brief, heart tissues from each rat were suspended in extraction buffer A homogenized at the highest setting for 2 min; and centrifuged at 1,000 g for 10 min at 4°C. Supernatants represent the cytosolic fraction. The pellets, containing enriched nuclei, were resuspended in buffer B. After centrifugation at 15,000 g for 30 min at 4°C, the supernatants containing the nuclear protein were stored at −80° C for further analysis. The levels of IKb-alpha were quantified in cytosolic fraction from heart tissue collected after I/R, whereas NF-κB p65 levels were quantified in nuclear fraction. The filters were blocked with 1x PBS, 5% (w/v) non-fat dried milk for 40 min at room temperature, and they were subsequently probed with specific antibodies anti-ikb-alpha, anti-NFkB p65 at 4°C overnight. Membranes were incubated with peroxidase-conjugated bovine antimouse IgG secondary antibody or peroxidase-conjugated goat antirabbit IgG for 1 h at room temperature. The relative expression of the protein bands of NF-κB p65 (~75 kDa), IKB-a (~35 kDa), were quantified as described before (Di Paola et al., 2016).

#### Enzyme-linked immunosorbent assay measurement of TNF-α and IL-1β

For the measurement of cytokines levels, a 1 cm sample containing the lesion site (or comparable region of sham-operated animals) was dissected and homogenized in 1 ml PBS containing protease inhibitors (Complete protease inhibitor tablets, Roche). TNF-α and IL-1β levels were assayed using DuoSet ELISA Development System (R&D Systems, Minneapolis, MN, USA). All assays were performed in duplicate using recommended diluents, buffers, and substrates. Absorbency was measured using a microplate reader at 450 nm (Multiskan FC Microplate Photometer, Thermo Fisher Scientific Inc., Rockford, IL, USA). The intra-assay coefficient of variations for both assays was less than 10%. The concentration of the cytokines in the tissue was mentioned as protein in pg/mg.

#### Data analyses

All values were expressed as mean ± standard error of the mean (SEM) of N observations. For the *in vivo* studies N represents the number of animals studied. In the experiments including histology or immunohistochemistry, at least three experiments were executed on different experimental days. The statistical results were obtained as assessed before (Esposito et al., 2011). We analyzed *n* = 5 slides from each sample collected from all animals in each experimental group. Non-parametric data were analyzed with the Fisher's exact test. A *p*-value less than 0.05 will be considered significant. ^*^p < 0.05 vs. Sham; °*p* < 0.05 vs. I/R.

## Results

### Effect of pistachios on myocardial infarct size extension

**Figure 1:**
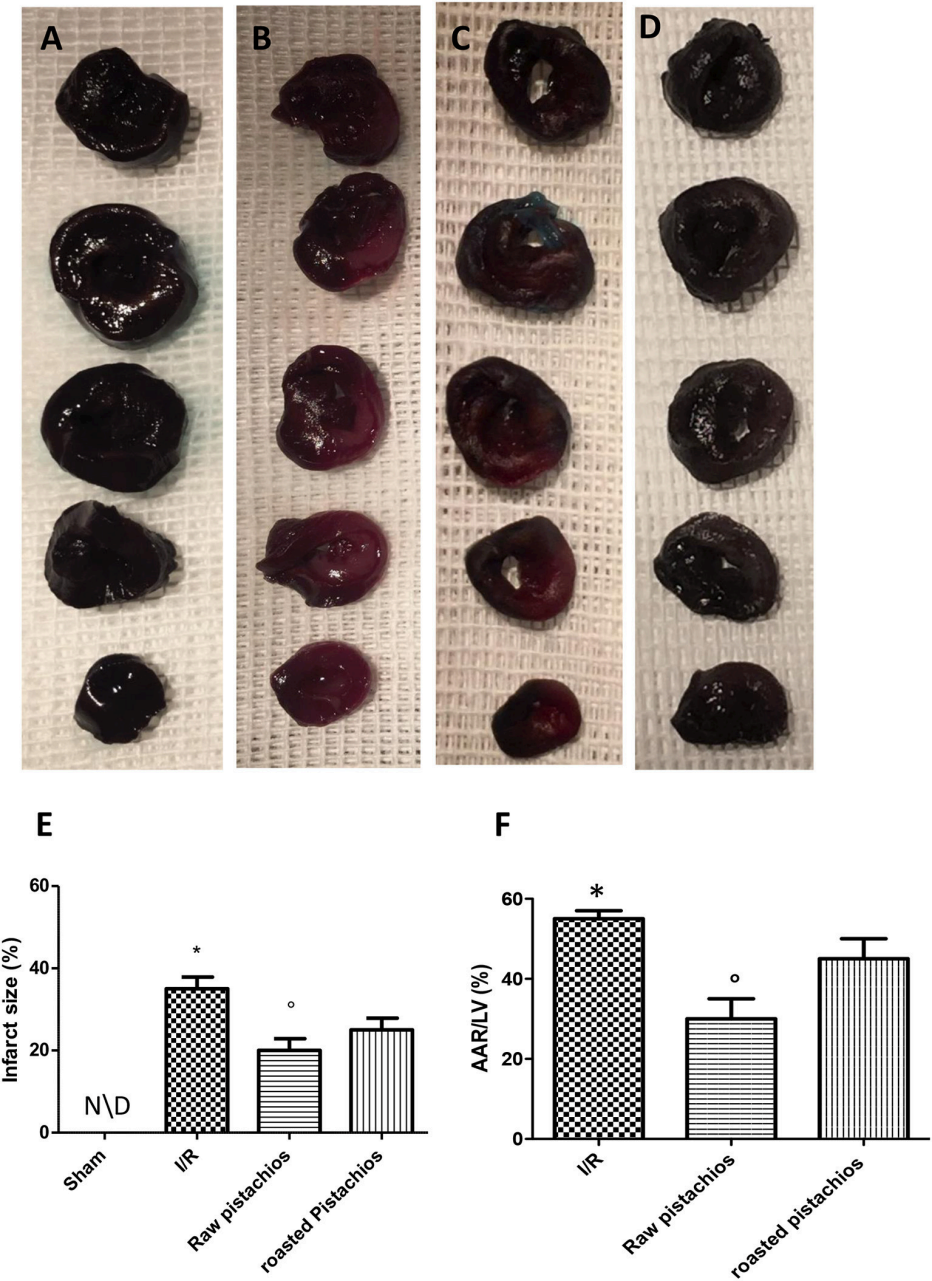
Effect of pistachios on infarct size and area at risk. In STZ-ischemia-reperfusion rats, myocardial ischemia/ reperfusion resulted in significantly increased infarct size **(B)** when compared with sham-operated animals in **(A)**. Blue stained region indicates non-ischemic, normal region; red-stained region indicates ischemic/reperfused but not infarcted region; and negative-stained region indicates ischemic/reperfused infarcted region. NP caused a significant reduction in myocardial infarct size when compared with vehicle-treated animals **(C)**. The effect of NP was higher compared with RP **(D)**. Quantification of myocardial infarct size expressed as percentage **(E)** and as percentage of area-at-risk(AAR) **(F)**. Data are means ± S.E.M. of 10 rats for each group. ^*^P < 0.05 vs. sham, °*P* < 0.05 vs. ischemia-reperfusion.

p-Nitroblue tetrazolium staining showed that myocardial damage was more extensive in ischaemic-reperfused hearts (Figures 1B,E,F). NP (30 mg/kg) reduced the extension of myocardial damage (Figures 1C,E,F). However, treatment with RP did not provide a significant reduction in infarct size (Figures 1D,E,F). No significant alteration was made by administration of pistachios in sham (Figures 1A,E,F). Moreover, treatments with NP differed significantly with RP in modifying the extension of infarct size, expressed as per cent of area at risk (AAR) of at risk area per left ventricle (LV) (Figure 1F); Sham-operation did not result in a significant degree of infarction (Figure 1A).

### Effect of pistachios on histological damage

**Figure 2:**
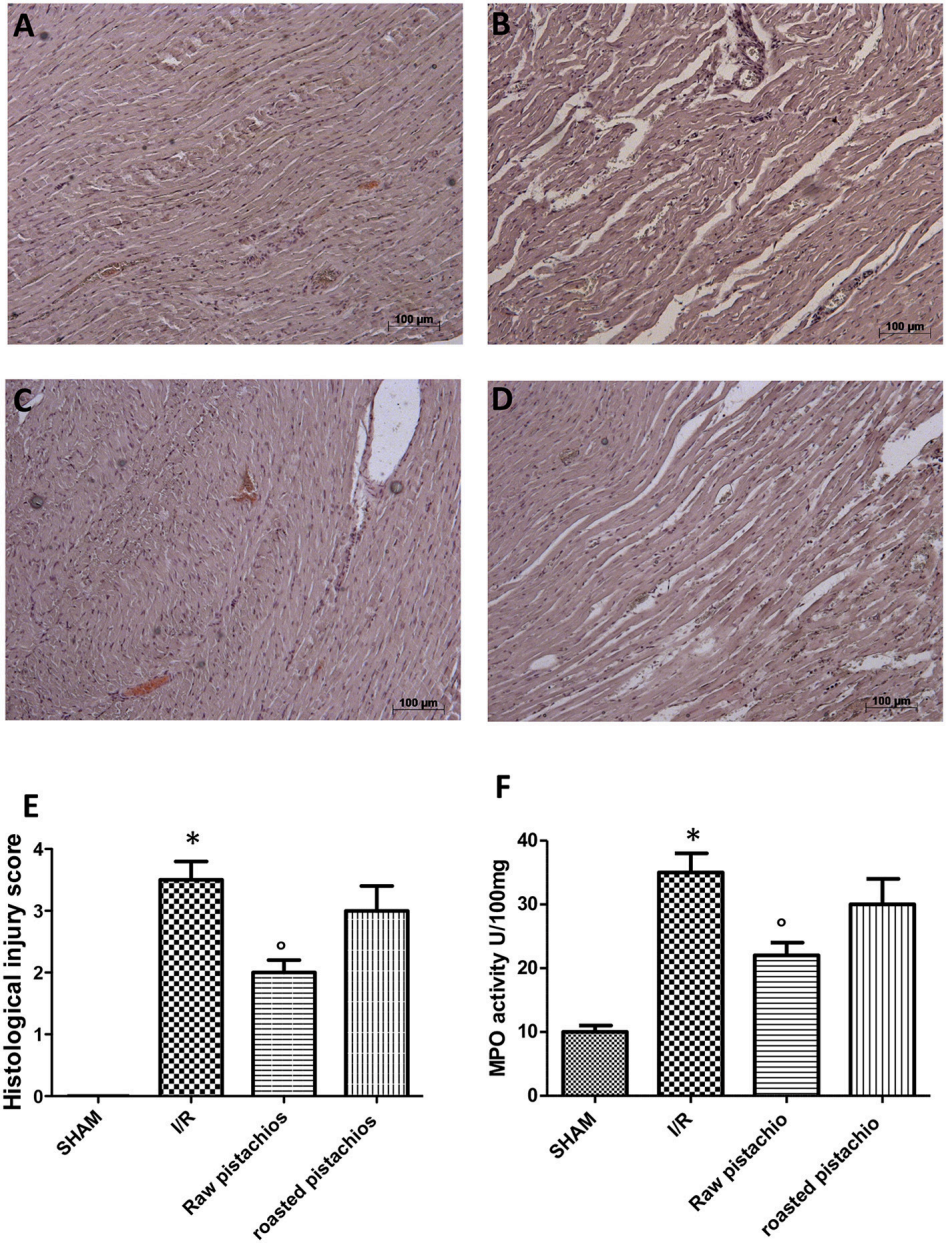
Effect of pistachios on ischemia-reperfusion injury. Histological examination (haematoxylin/eosin staining) showed tissue damage, necrosis with massive neutrophil infiltration in ischemic reperfused-heart **(B)** compared to sham rats whose histological myocardial structure was typical of a normal architecture **(A)**. NP (30 mg/kg) treatment **(C)** showed an important reduction of tissue damage compared to the STZ-I/R group **(B)**. RP provided a minor effect on tissue damage **(D)**. The histological score was made by independent observer **(E)**. Moreover neutrophil infiltration was significantly increased in STZ- myocardial I/R- rats in comparison to sham **(F)**. NP-treated rats showed an important decrease of neutrophil infiltration **(F)**. Treatment with RP did not provide a significant decrease in MPO activity **(F)**. Data are means ± S.E.M. of 10 rats for each group. ^*^P < 0.05 vs. sham, °*P* < 0.05 vs. ischemia-reperfusion.

Histological examination showed tissue damage, necrosis with massive neutrophil infiltration in ischemic reperfused-heart (Figure 2B, see histological score Figure 2E) compared to sham rats, whose histological myocardial structure was typical of a normal architecture (Figure 2A, see histological score Figure 2E). NP (30 mg/kg) treatment (Figure 2C, see histological Figure 2E) showed an important reduction of tissue damage compared to the STZ-I/R group (Figure 2B, see histological score Figure 2E). The effect of NP was higher compared to RP (Figures 2C,D, see histological score Figure 2E). In the heart tissues collected from rats subjected to I/R we observed an increase of MPO activity compared with sham-operated rats (Figure 2F). Administration of pistachios (30 mg/kg) significantly attenuated MPO activity (Figure 2F). As before, the effect of NP was higher compared with RP (Figure 2F). No significant alteration was made by administration of pistachios in sham (Figure 2F).

### Effect of pistachios on TNF-α and IL-1β

**Figure 3:**
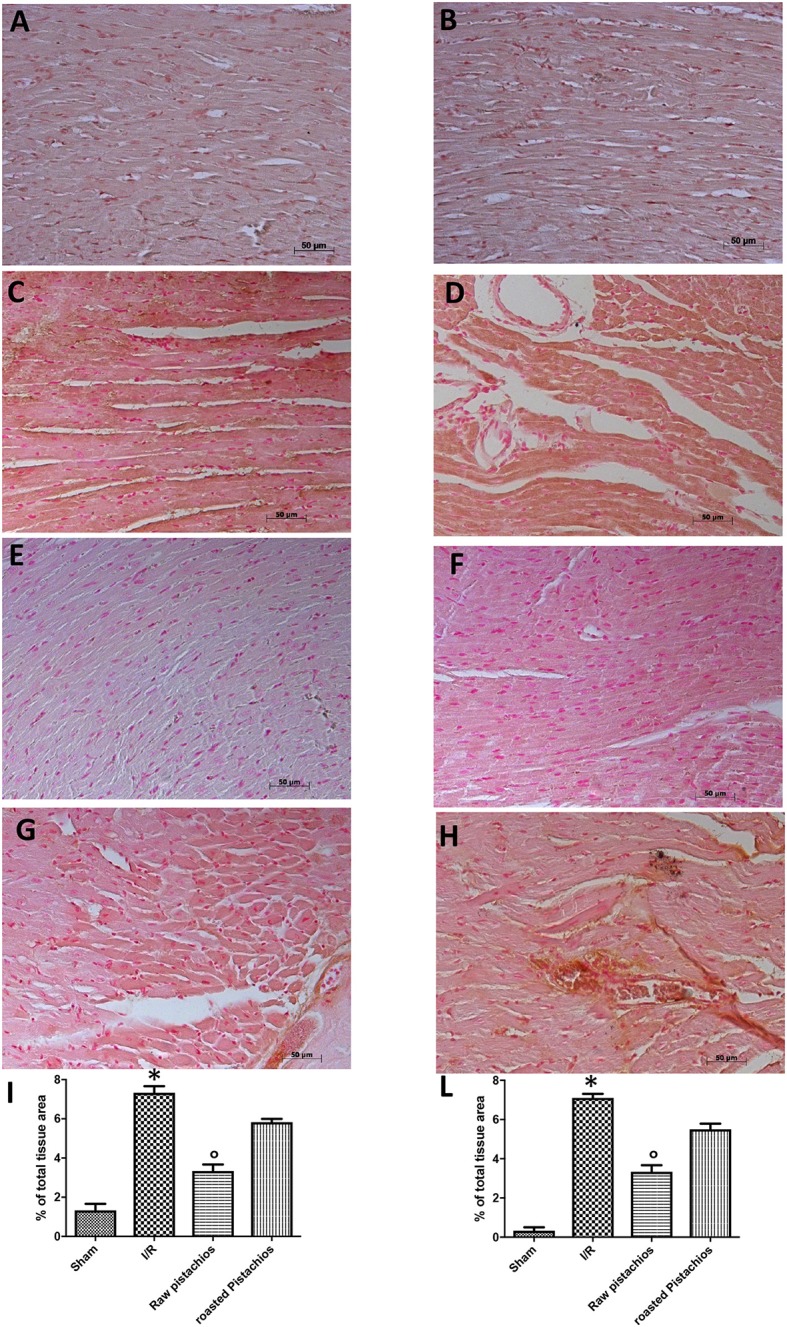
Effect of pistachios on cytokines production. The inflammatory response includes the expression of cytokines in the late stage of reperfusion. The tissue levels of TNF-α **(C)** and IL-1β **(D)** increased in STZ-I/R rats during the ischemia reperfusion injury, compared to the sham treated animals **(A,B)**. NP reduced tissue levels of TNF-α **(E)** and IL-1β **(F)**. RP provided a minor effect on TNF-α **(G)** and IL-1β **(H)** tissue expression. Densitometric analysis of immunocytochemistry photos (*n* = 5 photos from every sample obtained from each rats in all experimental group) for TNF-α **(I)** and IL-1β **(L)** from heart tissues was evaluated. Densitometric analysis was performed by using Optilab Graftek software on a Macintosh personal computer (CPU G3-266). Data are indicated as % of total tissue area. Data are means ± S.E.M. of 10 rats for each group. ^*^P < 0.05 vs. sham, °*P* < 0.05 vs. ischemia-reperfusion.

We analyzed the heart level of pro-inflammatory cytokines TNF-α and IL-1β. Positive staining for TNF-α (Figure 3C, see densitometry analysis Figure 3I) and for IL-1β (Figure 3D, see densitometry analysis Figure 3L) was principally located in the infiltrated inflammatory cells in damaged tissues. Treatment with NP significantly reduced the staining for TNF-α (Figure 3E, see densitometry analysis Figure 3I) and for IL-1β (Figure 3F, see densitometry analysis Figure 3L) in the heart tissues taken after 2 h of reperfusion. The effect of NP was higher compared with RP (Figures 3G,H see densitometry analysis Figures 3I,L). No positive staining for TNF-α and IL-1β was found in myocardial tissue section from sham-operated rats (Figures 3A,B see densitometry analysis Figures 3I,L) Also enzyme-linked immunosorbent assay displayed an increased concentration of TNF-α (Figure 10A) and IL-1β (Figure 10B) in tissue from vehicle treated animals, while NP administration reduced this concentration. The effect of NP was higher compared with RP. Basal expression was found in myocardial tissue from sham-operated rats (Figures 10A,B).

### Effect of pistachios on cytokines and inflammatory cell

**Figure 4:**
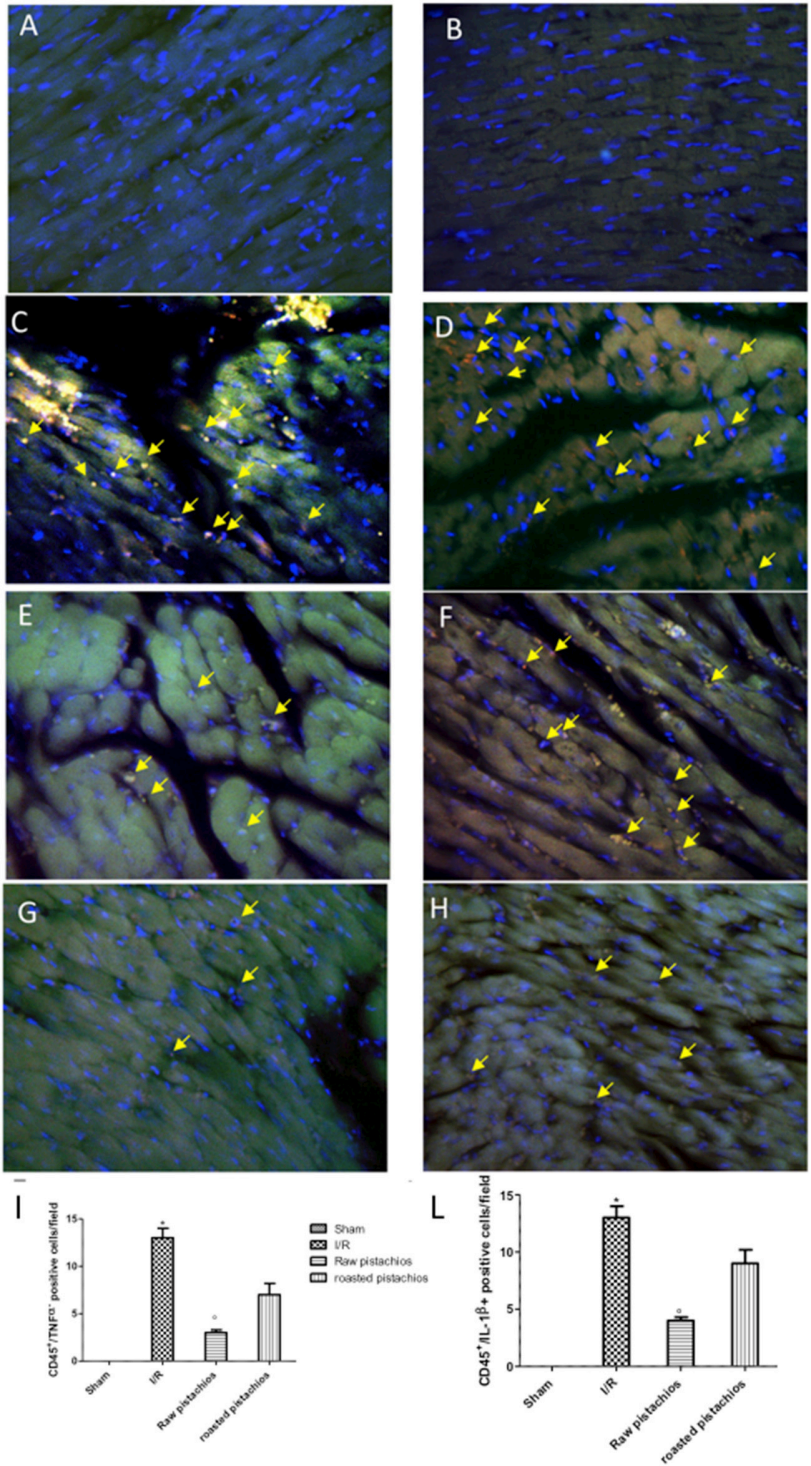
Effect of pistachios on cytokines co-localization with inflammatory cell marker. Positive co-staining of TNF-α **(C)** and IL-1β **(D)** with CD45 increased in STZ-I/R rats during the ischemia reperfusion injury. NP reduced the number of positive cells (**E,F** see densitometric analysis **I,L**). RP provided a minor effect on TNF-α (**G** see densitometric analysis **I**) and IL-1β (**H** see densitometric analysis **L**) co-localization with CD45. No positive cells were detected in sham treated animals **(A,B)**. The pictures are indicative of at least three experiments performed on different experimental days. Images are representative of all the mice in every group. A resolution of 8 bits was used to digitalize all images into an rank of 2048 x 2048 pixels. Data are means ± S.E.M. of 10 rats for each group. ^*^P < 0.05 vs. sham, °*P* < 0.05 vs. ischemia-reperfusion. The yellow arrow indicates positive cells where is detected the co-localization.

In order to evaluate the interaction between pro-inflammatory cytokines with inflammatory cell we performed immunofluorescence analysis of TNF-α and IL-1β and CD45, an inflammatory cell marker. Positive co-staining for TNF-α (Figure 4C, see densitometry analysis Figure 3I) and for IL-1β (Figure 4D, see densitometry analysis Figure 3L) with CD45 was principally located in the infiltrated inflammatory cells in damaged tissues. Treatment with NP significantly reduced the number of positive cells (Figure 4G, see densitometry analysis Figure 3I) (Figure 4H, see densitometry analysis Figure 3L) in the heart tissues taken after 2 h of reperfusion. The effect of NP was higher compared with RP (Figures 4E,F see densitometry analysis Figures 4I,L). No positive cells were found in myocardial tissue section from sham-operated rats (Figures 4A,B see densitometry analysis Figures 4I,L).

### Effect of pistachios on adhesion molecules expression

**Figure 5:**
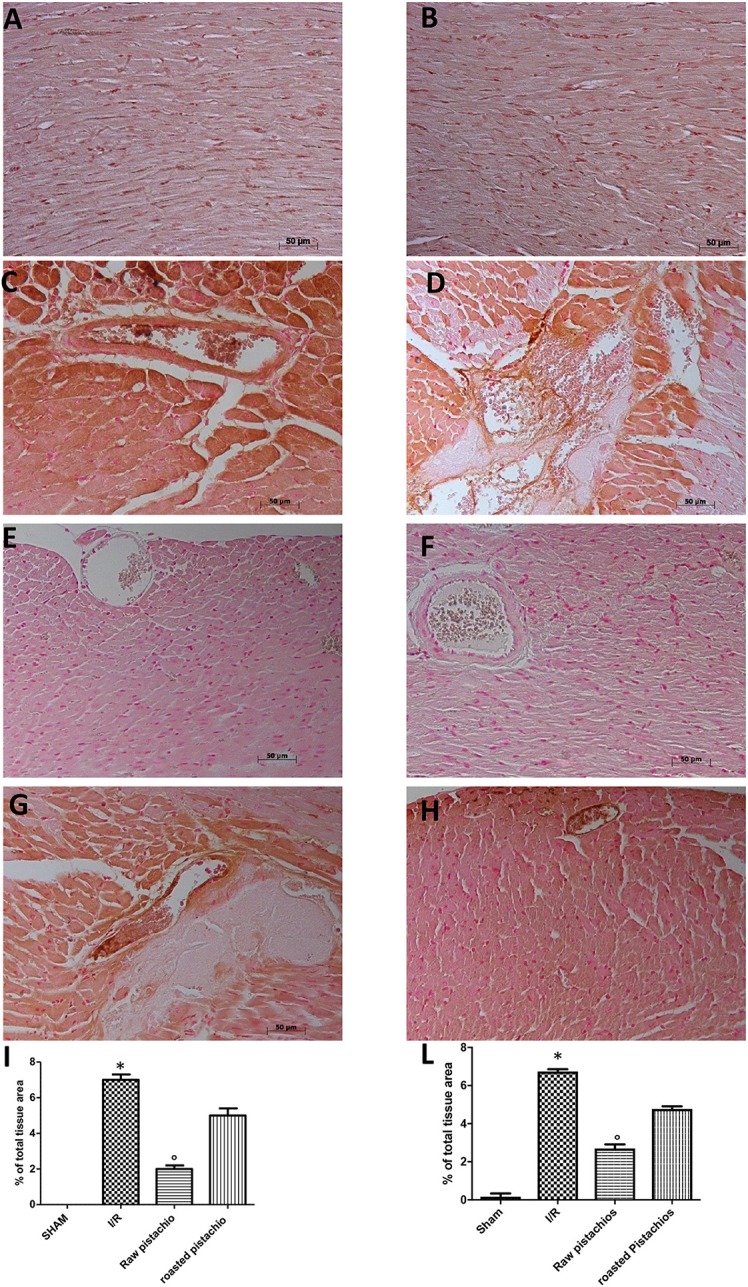
Effect of pistachios on adhesion molecules expression. Immunohistochemical analysis of myocardial sections obtained from STZ rats subjected to myocardial ischemia/reperfusion revealed a positive staining for ICAM-1 **(C)** and P-selectin **(D)** in the injured tissues, which was primarily localized around the vessel. There was no detectable immunostaining for ICAM-1 **(E)** and P-selectin **(F)** in the heart tissues obtained from rats treated with NP. Treatments with RP did not provide a significant reduction in ICAM-1 **(G)** and P-selectin **(H)** tissue expression. No positive staining was found in tissues collected from sham treated rats **(A,B)**. Densitometry analysis of immunocytochemistry photographs (*n* = 5 photographs from all samples collected from each rats in different groups) for ICAM-1 **(I)** and P-selectin **(L)** was assessed. The assay was carried out by Optilab Graftek software on a Macintosh personal computer (CPU G3-266). Data are indicated as % of total tissue area. This figure is expressive of at least 3 experiments executed on different experimental days. Data are means ± S.E.M. of 10 rats for each group. ^*^P < 0.05 vs. sham, °*P* < 0.05 vs. ischemia-reperfusion.

An increased imununohistochemical staining of adhesion molecules ICAM-1 (Figure 5C, see densitometry Figure 5I) and P-selectin, (Figure 5D, see densitometry Figure 5L) was found in the heart tissue sections obtained from I/R injured rats. NP visibly and significantly reduced the positive immunostaining for ICAM-1 (Figure 5E, see densitometry Figure 5I) and P-selectin (Figure 5F, see densitometry Figure 5L). The effect of RP on adhesion molecules expression was less positive (Figures 5G,H see densitometry Figures 5I,L). No positive immunostaining for adhesion molecules expression was found in heart tissue section obtained from sham-operated rats (Figures 5A,B see densitometry Figures 5I,L) Also immunofluorescence analysis confirmed this data. Positive co-staining for ICAM-1 with P-selectin (Figure 11C, see densitometry analysis Figure 11I) was found in damaged tissues. Treatment with NP significantly reduced the number of positive cells (Figure 11E, see densitometry analysis Figure 11I) in the heart tissues taken after 2 h of reperfusion. The number of positive cells was higher in RP compared with NP (Figure 11G see densitometry analysis Figure 11I). No positive cells were found in myocardial tissue section from sham-operated rats (Figure 11A see densitometry analysis Figure 11I).

### Effect of pistachios on nitrotyrosine formation, lipid peroxidation, and PARP activation

**Figure 6:**
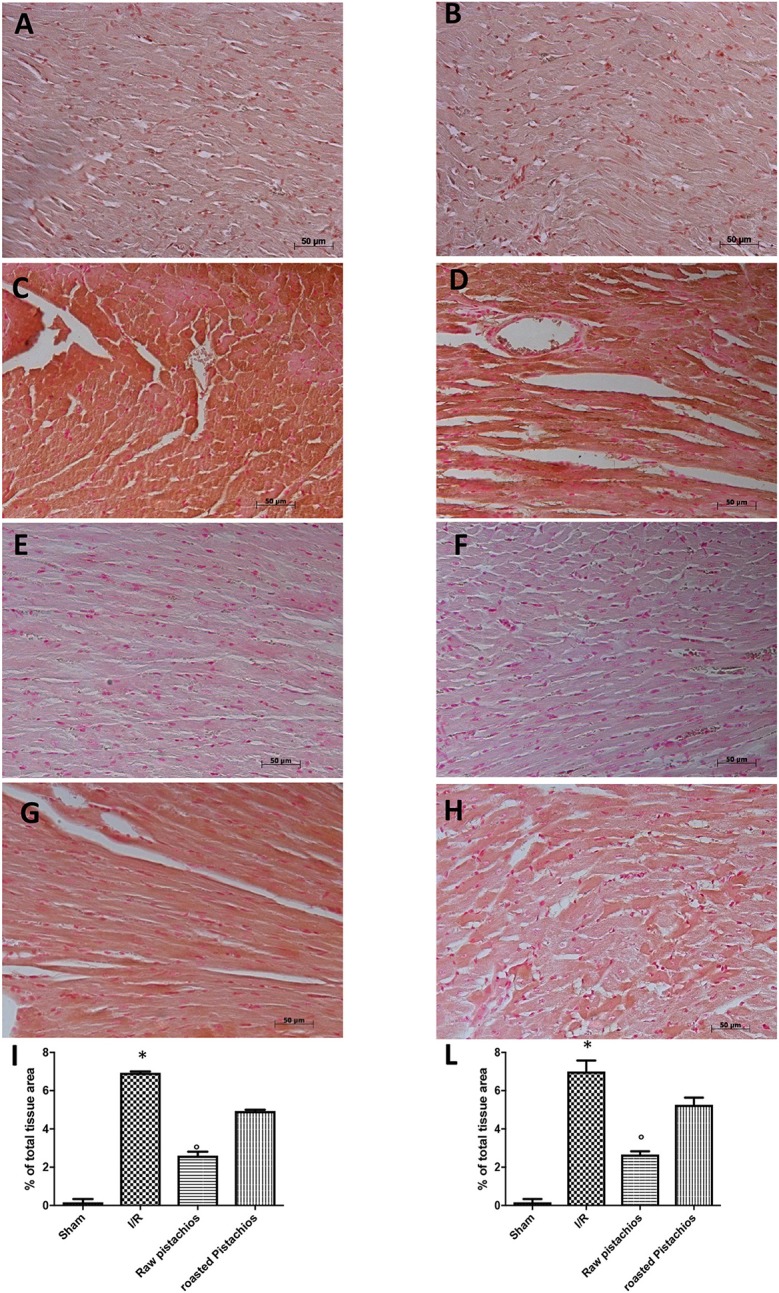
Effect of pistachios on nitrotyrosine and PAR. Immunohistochemistry for nitrotyrosine **(C)** and PAR **(D)** showed positive staining in the heart tissue section from STZ-I/R -treated rats, compared to the sham treated animals **(A,B)**. The intensity of the positive staining for nitrotyrosine **(E)** and PAR **(F)** was significantly reduced in the heart from natural raw pistachios-treated rats. Treatments with RP did not provide a significant reduction in nitrotyrosine production **(G)** and PAR **(H)**. Densitometry analysis of immunocytochemistry photographs (*n* = 5 photos from each sample collected from all rats in each experimental group) for nitrotyrosine **(I)** and PAR **(L)** from heart tissues was assessed. The assay was carried out by using Optilab Graftek software on a Macintosh personal computer (CPU G3-266). Data are expressed as % of total tissue area. This figure is representative of at least 3 experiments performed on different experimental days. Data are expressed as mean ± s.e.m. from *n* = 10 rats for each group. ^*^P < 0.05 vs. sham, °*P* < 0.05 vs. I/R.

Nitrotyrosine, a specific indicator of nitrosative stress, and PAR, an indicator of *in vivo* PARP activation, were measured by immunohistochemical analysis in the heart tissue sections of different groups, 2 h after reperfusion. A positive immunohistochemical staining for nitrotyrosine (Figure 6C, see densitometry Figure 6I) and PARP (Figure 6D see densitometry Figure 6L) was found in myocardial tissue sections collected from I/R STZ-rats. On the contrary, NP (30 mg/kg) reduced nitrotyrosine (Figure 6E, see densitometry Figure 6I) and PARP (Figure 6F, see densitometry Figure 6L) immunostaining. The effect of RP on nitrotyrosine (Figure 6G see densitometry Figure 6I) and PARP (Figure 6H, see densitometry Figure 6L) expression was less positive. No positive staining for nitrotyrosine and PARP was found in heart tissues obtained from sham-operated rats (Figures 6A,B see densitometry Figures 6I,L). Also immunofluorescence analysis confirmed this data. Positive co-staining for nitrotyrosine with PARP (Figure 11D, see densitometry analysis Figure 11L) was found in damaged tissues. Treatment with NP significantly reduced the number of positive cells (Figure 11F, see densitometry analysis Figure 11L) in the heart tissues taken after 2 h of reperfusion. The number of positive cells was higher in RP compared with NP (Figure 11H see densitometry analysis Figure 11L). No positive cells were found in myocardial tissue section from sham-operated rats (Figure 11B see densitometry analysis Figure 11L).

### Effect of pistachios on apoptosis

**Figure 7:**
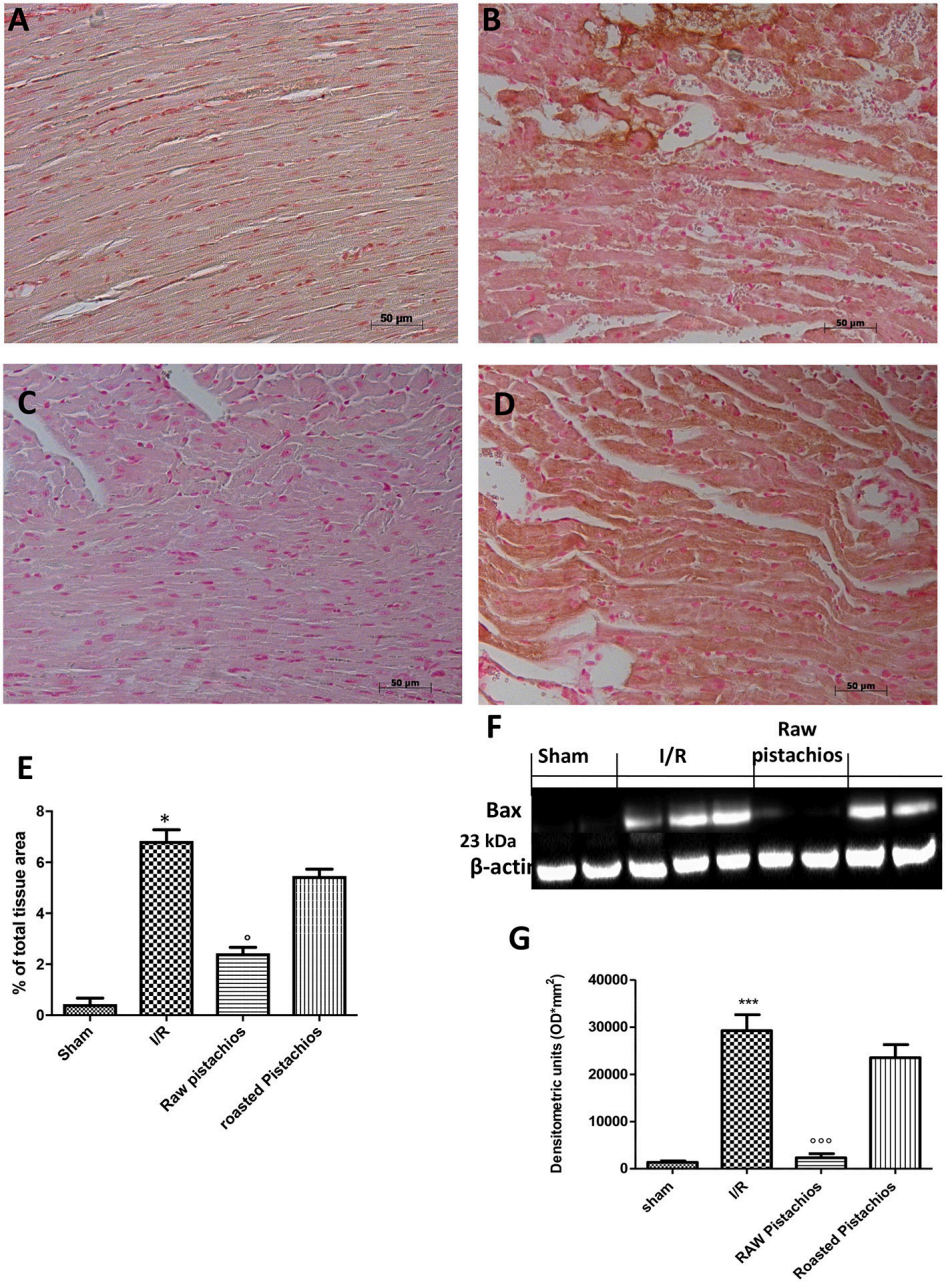
Effect of pistachios treatments BAX expression. Representative Western blots showed basal level of BAX in heart tissues obtained from sham-treated animals **(F,G)**. Bax levels were increased in the heart tissues from I/R-treated rats **(F,G)**. NP prevented the I/R-induced Bax expression **(F,G)**. Treatments with RP did not provide a significant reduction in Bax expression **(F,G)**. Bax levels was evaluated by immunohistochemistry, in the same way NP significantly reduced bax positive staining **(C)** when compared with STZ-IR treated rats **(B)**. Treatment with RP did not provide a significant reduction in Bax immunohistochemical staining **(D)**. No expression of BAX was found in tissue from sham treated rats **(A)**. A representative blot of lysates obtained from 5 animals per group is shown and densitometry analysis of all animals is reported. The results are expressed as mean ± S.E.M. from *n* = 5/6 heart tissues for each group. Densitometry analysis of immunocytochemistry photographs (*n* = 5 photos from each sample collected from all rats in each experimental group) BAX **(E)** from heart tissues was assessed. The assay was carried out by using Optilab Graftek software on a Macintosh personal computer (CPU G3-266). Data are expressed as % of total tissue area. This figure is representative of at least 3 experiments performed on different experimental days. Data are expressed as mean ± S.E.M. from *n* = 10 rats for each group. ^*^P < 0.05 vs. sham, °*P* < 0.05 vs. I/R, ^***^P < 0.001 vs. sham, °°°*P* < 0.001 vs. I/R.

**Figure 8:**
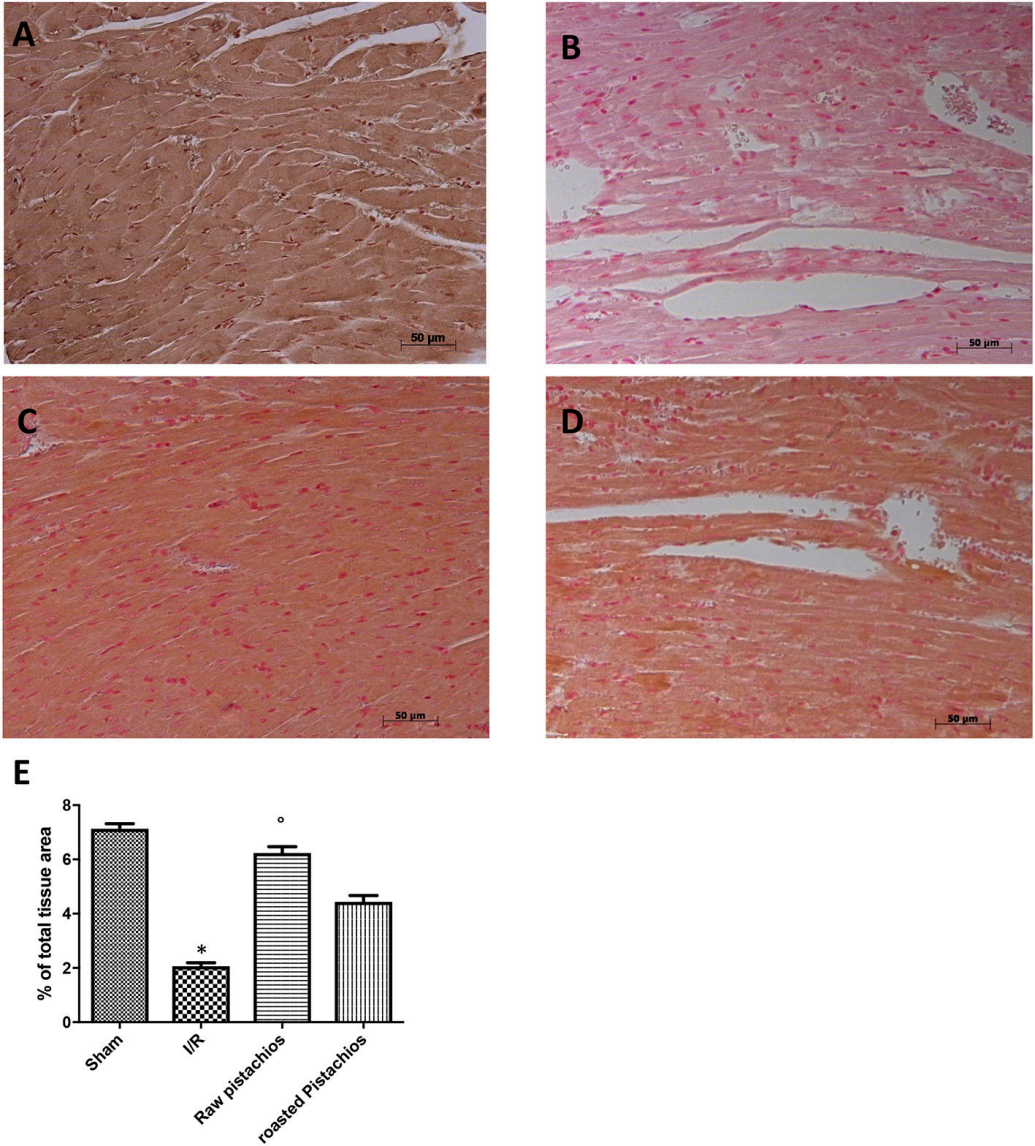
Effect of pistachios treatments Bcl-2 expression. Heart tissues collected from sham treated animals displayed basal expression of Bcl-2 **(A)**, while Bcl-2 expression was significantly reduced in the heart tissues from I/R-treated rats **(B)**. Treatment NP significantly attenuated I/R shock-induced inhibition of Bcl-2 expression **(C)**. The effect of RP on Bcl-2 **(D)** was less positive. Densitometry analysis of immunocytochemistry photographs (*n* = 5 photos from each sample collected from all rats in each experimental group) Bcl-2 **(E)** from heart tissues was assessed. The assay was carried out by using Optilab Graftek software on a Macintosh personal computer (CPU G3-266). Data are expressed as % of total tissue area. This figure is representative of at least 3 experiments performed on different experimental days. Data are expressed as mean ± S.E.M. from *n* = 10 rats for each group. ^*^P < 0.05 vs. sham, °*P* < 0.05 vs. I/R.

Heart samples were collected 2 h after reperfusion in order to evaluate the immunohistological staining for Bax and Bcl-2. Heart samples collected from sham-treated rats did not show a positive Bax staining (Figure 7A see densitometry Figure 6E), whereas heart tissues obtained from I/R- STZ rats exhibited positive staining for Bax (Figure 7B see densitometry Figure 6E). NP reduced the Bax positive staining in the heart sections of rats subjected to I/R (Figure 7C see densitometry Figure 6E). In addition, heart sections from sham-treated rats demonstrated positive staining for Bcl-2 (Figure 8A see densitometry Figure 8E) whereas Bcl-2 staining was significantly reduced (Figure 8B see densitometry Figure 8E) in I/R treated rats. NP significantly attenuated the loss of positive staining for Bcl-2 in rats subjected to I/R (Figure 8C see densitometry Figure 8E). The effect of RP on Bax (Figure 7D see densitometry Figure 7E) and Bcl2 (Figure 8D see densitometry Figure 8E) expression was less positive compared to NP. The presence of Bax in heart homogenates was also investigated by Western blot. No Bax expression was detected in heart tissues obtained from sham-operated animals (Figures 7F,G). Bax levels were higher in the heart tissues from I/R- rats (Figures 7F,G). On the contrary, NP prevented the I/R-induced Bax expression (Figures 7F,G). The effect of RP on Bax (Figures 7F,G) expression was less positive compared to NP.

### Effect of pistachios on activation of the NF-κB pathway

**Figure 9:**
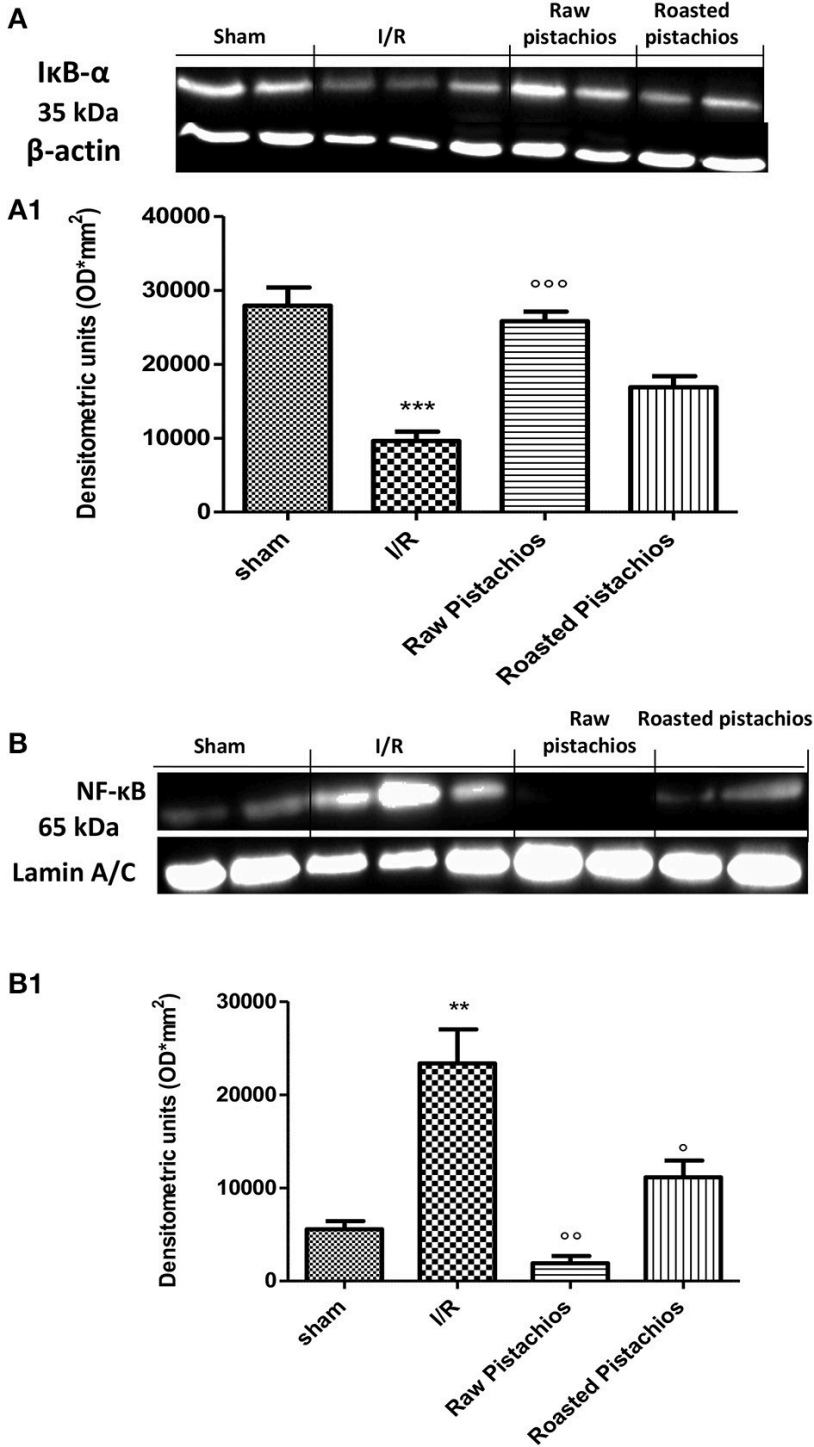
Effect of pistachios on IκB-α degradation and NF-κB expression. By Western blot analysis, a basal level of IκB-α was detected in the heart tissue from sham-operated animals **(A,A1)**, whereas in I/R control rats, IκB-α levels were substantially reduced **(A,A1)**. NP treatment prevented the I/R-induced IκB-α degradation **(A,A1)**. The effect of RP on IKB-alpha was less positive. Moreover, the levels of the NF-κB p65 subunit protein in the nuclear fractions of the heart tissue were also significant increased compared with the sham-operated rats **(B,B1)**. The levels of NF-κB p65 protein were significantly reduced in the nuclear fractions of the heart tissues from animals receiving NP as shown in **(B)**. Treatments with RP did not provide a significant reduction in NF-κB p65 protein levels **(B)**. A representative blot of lysates obtained from 5 animals per group is shown and densitometry analysis of all animals is reported. The results are expressed as mean ± S.E.M. from *n* = 5 heart tissues for each group. The results are expressed as mean ± S.E.M. from five blots. ^*^P < 0.05 vs. sham, °*P* < 0.05 vs. I/R.

In order to examine the cellular pathway by which treatment with pistachios may attenuate the progress of I/R, we assessed IκB-degradation, and NF-κB p65 levels by Western blot analysis. Treatment with NP (30 mg/kg) significantly reduced the I/R-induced IκB-α degradation (Figures 9A,A1). The effect was greater than RP. A physiological level of IκB-α was found in the sham myocardial tissue. Treatment with NP (30 mg/kg) reduced the levels of NF-κB p65 protein (Figures 9B,B1), whereas RP resulted in minor reduction in NF-κB levels (Figures 9B,B1).

**Figure 10:**
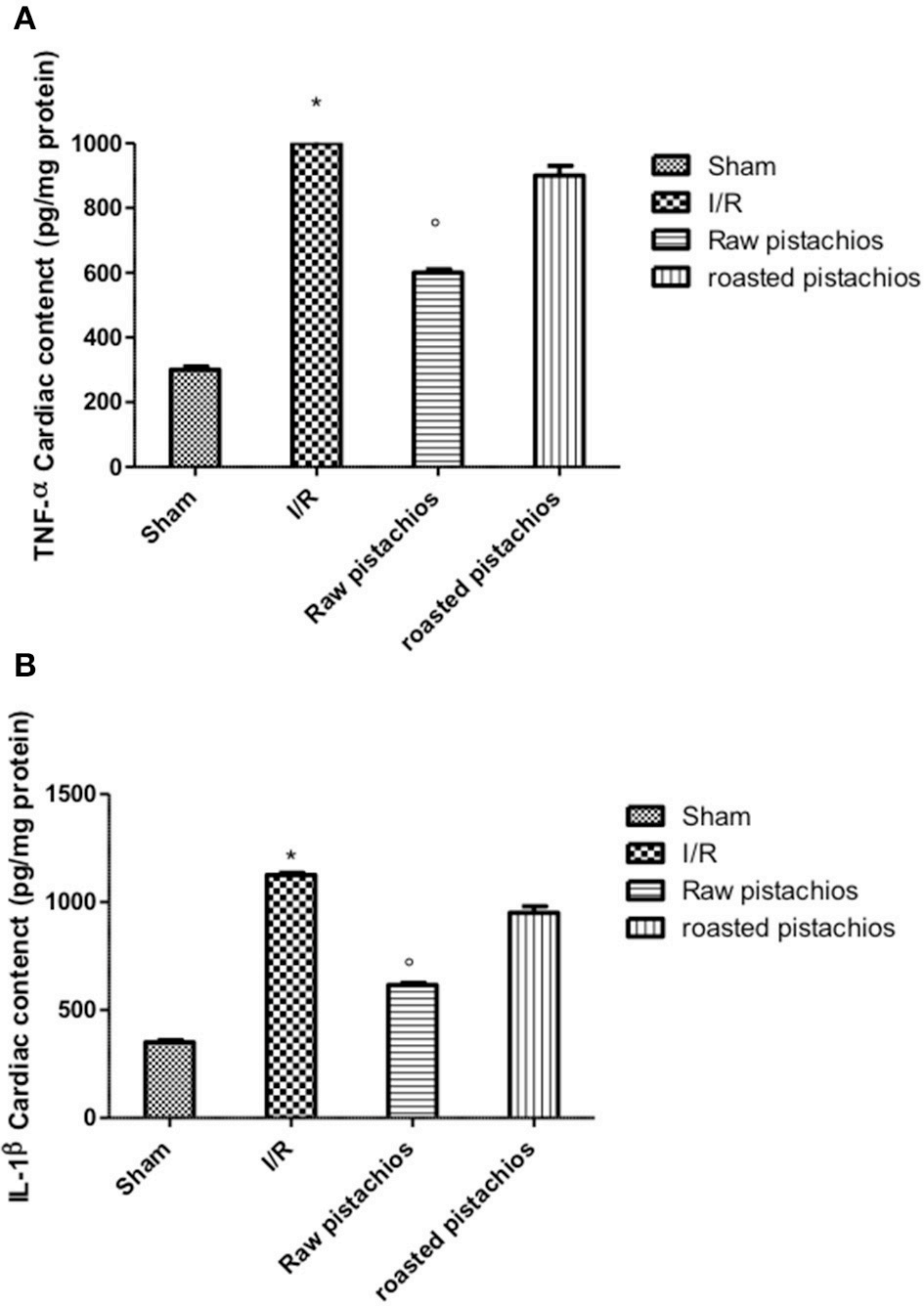
Effect of pistachios on cytokines concentration. Ischemia reperfusion injury caused an increase in the release of TNF-α **(A)** and IL-1β **(B)** in heart tissues. Treatment with NP significantly reduced TNF-α and IL-1β concentration, while RP had minor effect on this inhibition. Basal expression of this cytokines was found in tissues collected from sham-operated animals **(A,B)**. ^*^P < 0.05 vs. sham, °*P* < 0.05 vs. I/R.

**Figure 11:**
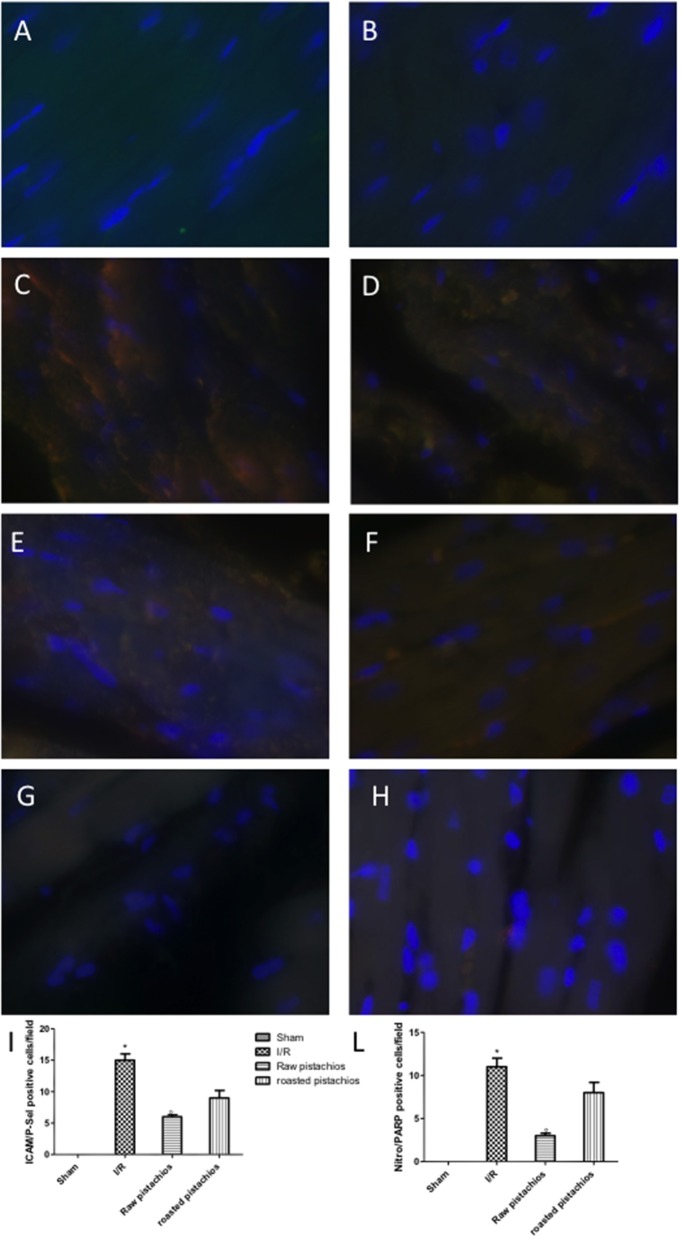
Effect of pistachios on adhesion molecules and nitrotyrosine and PARP co-localization. Positive co-staining of ICAM with P-selectin and nitrotyrosine with PARP increased in STZ-I/R rats during the ischemia reperfusion injury **(C,D)**. NP reduced the number of positive cells (**E,F** see densitometric analysis **I,L**). RP provided a minor effect on co-localization of ICAM with P-selectin (**G,H** see densitometric analysis **I,L**). No positive cells were detected in sham treated animals **(A,B)**. The pictures are indicative of at least three experiments performed on different experimental days. Images are representative of all the mice in every group. A resolution of 8 bits was used to digitalize all images into an rank of 2048 × 2048 pixels. Data are means ± S.E.M. of 10 rats for each group. ^*^P < 0.05 vs. sham, °*P* < 0.05 vs. ischemia-reperfusion.

## Discussion

In this study we demonstrated that treatment with pistachios reduced the inflammatory process related to myocardial ischemia reperfusion injury in diabetic rats. The beneficial effect of pistachios may be attributed to the presence of bioactive complexes, such as procyanidins and carotenoids. In particular, the higher effect observed with NP vs. RP could be ascribed to the concentration and distribution of polyphenolic compounds, as previously reported (Mandalari et al., 2013). Hyperglycaemia-dependent myocardial oxidative injury could be increased by consequent inflammatory process (Pieme et al., 2017; Yao et al., 2017). This concept was confirmed by animal studies showing increased levels of proinflammatory cytokines (IL-6, IL-1, TNF-α, IL-18) and peroxynitrite (an index of oxidative stress) in the myocardium of diabetic mice. They are related with the blood glucose levels (Silaghi et al., 2015) and finally lead to myocardial apoptosis and greater infarct size. Our results confirmed an increase of cytokines levels in myocardial tissues of diabetic rats subjected to I/R injury, in particular TNF-α and IL-1β, which are not constitutively expressed in the heart muscle. Treatment with natural raw pistachios provided significantly reduction in both cytokines levels. Roasted salted pistachios treatment showed a minor effect on TNF-α and IL-1β tissue expression. The adhesion molecules expression and inflammatory cytokines production are initial events after ischemia and reperfusion. Upon cytokines stimulation, such as tumor necrosis factor (TNF) and interleukin-1 (IL-1), the adhesion molecules concentration considerably increases, and they are expressed in the vascular endothelium, macrophages, and lymphocytes (Jing et al., 2014). Numerous cell adhesion molecules are identified to have critical roles in the cell surface interactions between PMNs and endothelial cells. One of the best-known adhesion molecules located on the endothelium surface is intercellular cell adhesion molecule 1 (ICAM-1). Hyperglycaemia upregulates the expression of cell adhesion molecules on the endothelium, a process recognized to induce pathological leukocyte-endothelium interactions. We observed here that ICAM-1 levels significantly increased in heart tissues after 2 h of myocardial ischemia reperfusion, suggesting ischemia induces inflammatory responses in the heart. Treatment with RP did not provide a significant reduction in ICAM-1 and P-selectin tissue expression, whereas NP significantly reduced adhesion molecules expression. PMNs accumulate in ischemic and reperfused myocardium under the stimulus of chemoattractants, and possess the capacity to produce oxygen-derived free radicals (ROS) when activated by appropriate stimuli (Frangogiannis, 2008). Our results clearly demonstrated an increase in MPO (a peroxidase enzyme abundantly expressed in neutrophil granulocyte) activity in STZ- myocardial I/R- rats in comparison to sham, NP-treated rats showing an important decrease of neutrophil infiltration. Treatments with RP did not provide a significant decrease in MPO activity. Reactive oxygen species (ROS) have a crucial role in priming the post-infarction inflammatory answer. ROS react right with cellular lipids, proteins and DNA, producing cell injury and death. It appears that hyperglycaemia *per se* can induce oxidative stress and new interesting data come from studies of hyperglycaemic oxidative stress and nitrotyrosine in the heart (Matyas et al., 2016). Nitrotyrosine formation has been found during acute hyperglycaemia in working rat hearts during hyperglycaemia (Matyas et al., 2016). Acute exposure to high glucose increases both O2 and NO production. The interface of O_2_ with NO is very quick triggering to production of the potent oxidant peroxynitrite (Matyas et al., 2016). In this study we confirmed the nitrotyrosine expression in heart samples of I/R treated mice. NP significantly reduced nitrotyrosine expression but RP did not. Peroxynitrite causes DNA oxidation, resulting in consequent dimerization and activation of PARP. Poly (ADP) synthetase (PARS) is a protein altering and nucleotide-polymerizing enzyme which is copiously present in the nucleus. Here, we showed that NP only diminishes the increase in PARP activity caused by myocardial I/R. It has been shown that activation of the NF-κB system in the ischemic myocardium is responsible for the activation of different proinflammatory mediators (Lenardo and Baltimore, 1989; Misra et al., 2003). NF-κB is stimulated by several agents, including cytokines (such as Interleukin (IL)-1β and Tumor Necrosis Factor (TNF)-α) and ROS. Although these studies display an injurious role of NF-κB stimulation in the infarcted heart, other researches have shown that the NF-κB pathway should also mediate cytoprotective responses (Karin and Lin, 2002). Transgenic mice harboring cardiac-restricted level of a mutated IkBα protein that avoids nuclear translocation of NF-κB in cardiac myocytes had larger infarcts and considerably enhanced myocyte apoptosis in an experimental model of permanent ischemia (Misra et al., 2003). Our results confirmed that NF-κB is activated in heart tissue of mice after ischemia reperfusion and at the same time the administration of natural raw pistachios has significantly reduced the expression. Recent studies reported that myocardial ischemia and reperfusion injury stimulates myocardial apoptosis *in vivo* (Fliss and Gattinger, 1996) in particular in diabetics animals. It has also been demonstrated that the incidence of apoptosis increases in the heart of diabetics subjects (Fliss and Gattinger, 1996; Misra et al., 2003). In the present study we showed that treatment with NP significantly reduced BAX expression while it increased the protein values of Bcl-2. The effect of RP on BAX and Bcl2 was less positive compared to NP. Finally, our results show that NP had greater effect on the inflammatory process associated with myocardial ischemia than RP. In conclusion this study describes how pistachios can modulate the inflammatory process, associated with myocardial ischemia reperfusion injury, in diabetic animals.

## Author contributions

RDP: gave substantial contributions to the conception and design of the work and approved the version to be published; RF and EG: performed the experiments and approved the version to be published; RDA, MC: analyzed data and approved the version to be published. SL and AC: drafted the work and approved the version to be published; GM and SC: revised it critically for important intellectual content and approved the version to be published.

### Conflict of interest statement

The authors declare that the research was conducted in the absence of any commercial or financial relationships that could be construed as a potential conflict of interest.

## Abbreviations

AAR: Area at risk
Bax: bcl-2-like protein 4
Bcl-2: B-cell lymphoma 2
EDTA: Ethylenediaminetetraacetic acid
EGTA: ethylene glycol tetraacetic acid
HCl: chloridric acid
HE: hematoxylin and eosin
ICAM-1: Intercellular Adhesion Molecule 1
IL-1β: interleukin 1 beta
IκB-α: nuclear factor of kappa light polypeptide gene enhancer in B-cells inhibitor alpha
I/R: ischemia/reperfusion
LAD: left anterior descending
NaCl: sodium chloride
NF-κB: nuclear factor kappa B
NO: nitric oxide
(O2•^−^): superoxide anions
NP: natural raw
PAR: poly ADP-ribose
PBS: phosphate buffer saline
PMFS: Phenylmethylsulfonyl fluoride
ROS: reactive oxygen species
RP: roasted salted
STZ: streptozotocin
TNF-α: tumor necrosis factor alpha.
